# Fatal disseminated mucormycosis due to *Cunninghamella bertholletiae* infection after ABO-incompatible living donor liver transplantation: a case report

**DOI:** 10.1186/s40792-022-01516-4

**Published:** 2022-09-02

**Authors:** Atsuyoshi Mita, Shohei Hirano, Takeshi Uehara, Kai Uehara, Yasunari Ohno, Koji Kubota, Yuichi Masuda, Tsuyoshi Notake, Kazuki Yoshizawa, Akira Shimizu, Yuji Soejima

**Affiliations:** 1 Division of Gastroenterological, Hepato-Biliary-Pancreatic, Transplantation and Pediatric Surgery, Department of Surgery, Shinshu University School of Medicine, 3-1-1 Asahi, Matsumoto, 390-8621 Japan; 2 Department of Pathology, Shinshu University School of Medicine, Matsumoto, Japan

**Keywords:** Mucormycosis, Mycoses, *Cunninghamella bertholletiae*, Liver transplantation, Endocarditis

## Abstract

**Background:**

Fungal infection may develop because of immunosuppression after organ transplantation, in which invasive types, such as *Aspergillus* and *Mucorales*, fungi cause morbidity. We present a case of disseminated mucormycosis due to *Cunninghamella bertholletiae* after ABO-incompatible living donor liver transplantation (LDLT).

**Case presentation:**

A 47-year-old man with decompensated liver cirrhosis and hepatocellular carcinoma underwent an ABO-incompatible LDLT using a graft procured from his son, who had a different blood type. Rituximab and mycophenolate mofetil were administered 3 weeks before LDLT as immunosuppressive therapy. Although liver graft function improved, mass-like infiltrates appeared in the lungs following intubation for > 1 week due to impaired consciousness. The brain magnetic resonance imaging findings were normal. Decreased ejection fraction and ST elevation were detected on echocardiography and electrocardiography, respectively. There was no dominant stenosis on coronary arteriography. The recipient underwent segmentectomy of the right lung 20 days after LDLT. *C. bertholletiae* was identified from a specimen using polymerase chain reaction, thus establishing a diagnosis of mucormycosis. Multiple infarctions in the brain, heart, and kidney developed within 2 weeks. Treatment with amphotericin B was ineffective. The patient developed circulatory collapse, and a temporary pacemaker and percutaneous coronary intervention were required for cardiac infarction. The recipient died of cardiac failure 27 days after the LDLT. Autopsy revealed disseminated mucormycosis involving the brain, thyroid, heart, lung, liver, gastrointestinal tract, and both kidneys. In addition, fungal endocarditis may have been responsible for septic emboli in multiple organs, resulting in multiple organ invasion. Hypothrombocytopenia was present since the pre-transplant period, and the recipient was diagnosed posthumously with myelodysplastic syndrome due to hereditary abnormalities. Multiple factors such as organ transplantation, bone marrow dysfunction, immunosuppression, and inadequate administration of antifungal reagents might have promoted mucormycosis development in our patient.

**Conclusions:**

Mucormycosis by *C. bertholletiae* is a fatal complication; thus, early diagnosis and treatment are warranted before multiple organ invasion.

## Background

Fungal infections develop in 20–42% of liver transplant recipients due to immunosuppression after liver transplantation [1]. Invasive fungi, such as *Aspergillus* and *Mucorales* fungi, cause mortality in over 90% and 60% of liver transplant recipients, respectively [2, 3]. Mucormycosis is characterized by extensive angioinvasion. In particular, infections caused by *Cunninghamella bertholletiae* have significantly higher mortality rates than those of other *Mucorales* [4–6]. We present a case of disseminated mucormycosis due to *C. bertholletiae* after ABO-incompatible living donor liver transplantation (LDLT).

## Case presentation

A 47-year-old man with diabetes mellitus, decompensated liver cirrhosis due to non-alcoholic steatohepatitis, and hepatocellular carcinoma (HCC) underwent LDLT with splenectomy using a right hemiliver graft procured from his son. His pre-transplant model for end-stage liver disease score was 18 points, indicating that he was a suitable (not too severe) candidate for liver transplantation. Preoperative culture screening and β-d glucan were negative. The HCC lesion was 2 cm in diameter within the Milan criteria.

**Fig. 1:**
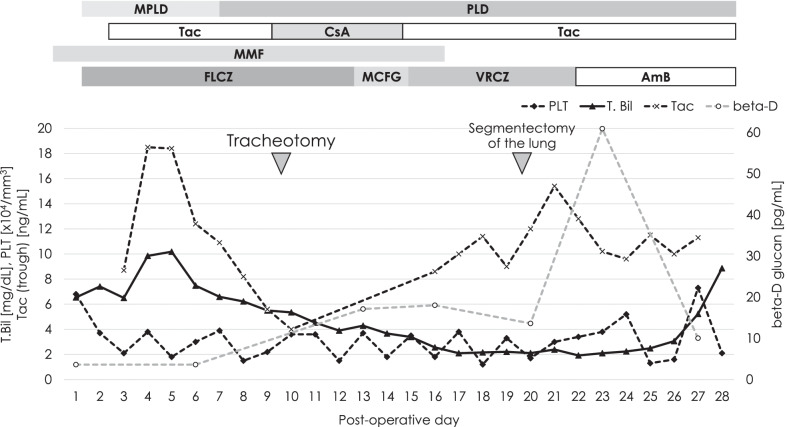
Post-transplant course. *AmB* amphotericin B, *FLCZ* fluconazole, *CsA* cyclosporine, *MCFG* micafungin, *MMF* mycophenolate mofetil, *MPLD* methylprednisolone, *PLD* prednisolone, *PLT* platelet, *Tac* tacrolimus, *T. Bil* total bilirubin, *VRCZ* voriconazole

This was a case of ABO-incompatible LDLT from a type-B-positive donor to an O-positive recipient. The patient was administered intravenous rituximab at a dose of 375 mg/m^2^ 3 weeks before the LDLT and oral mycophenolate mofetil (MMF) 2 g/day starting 2 weeks prior to the LDLT for induction immunosuppression. Post-transplant immunosuppression comprised tacrolimus, MMF, and steroids. The patient received oral fluconazole (100 mg/day) for 10 days to prevent fungal infections before switching to micafungin. The trough level of tacrolimus was adjusted by daily measurement, targeting a concentration of 10 ng/mL (Fig. 1). Post-transplant surveillance to detect fungal infection consisted of chest X-ray twice a day, culture screening of the sputum sample twice a week, and β-d glucan testing once a week.

The patient’s liver graft function gradually improved, and serum bilirubin levels decreased postoperatively (Fig. 1). After the LDLT, the patient’s level of consciousness remained impaired, with a Glasgow Coma Scale of G3V1M5, and he required intubation for > 1 week without respiratory failure. His oxygen saturation was maintained > 98% at 30% fraction of inspired oxygen. Brain magnetic resonance imaging was unremarkable. We temporally switched his tacrolimus to cyclosporine due to suspected adverse effects. However, there was no improvement in his consciousness. He underwent tracheotomy on postoperative day (POD) 9.

**Fig. 2:**
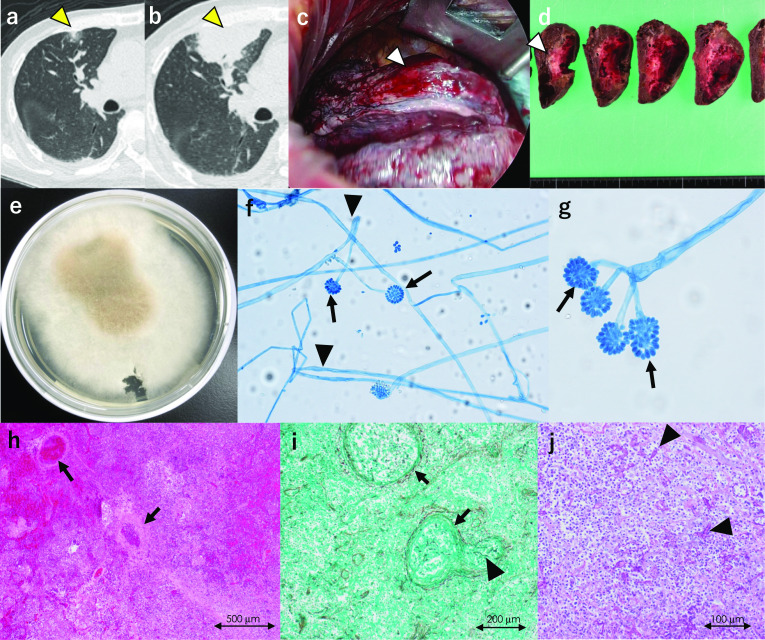
Lung lesion. **a**, **b** Pulmonary CT scan images show a small ground-glass nodule in the right upper lobe of the lung on POD 5 (**a**, yellow arrowhead) and that the size of the nodule gradually increased to 57 × 29 × 33 mm on POD 16 (**b**, yellow arrowhead). **c**, **d** Thoracoscopy identifies the nodule with subserous bleeding in the right upper lobe (segment 3) of the lung (**c**, white arrowhead), and the specimen obtained by subsegmentectomy consists of necrosis with bleeding but without abscess formation (**d**, white arrowhead). **e** Two-day culture from the specimen forms a cotton-like fungus on the plate of Sabouraud dextrose agar. **f**, **g** Microscopic findings reveal broad, aseptate hyphae (arrows) with wide-angle branching (arrowheads), suggesting mucor infection. **h**–**j** Alveolar tissue pathologically includes multiple bleeding necrosis foci with fungal emboli (arrows) in most arteries (hematoxylin and eosin stain). Arteries, including small capillaries, are fully filled by emboli (arrows) surrounding bleeding necrosis. These emboli comprise red blood cells and hyphae (arrowheads), stained with Grocott (**i**) and periodic acid–Schiff (**j**). *CT* computed tomography, *POD* postoperative day

Chest radiography showed a right mid-zone infiltrate (Fig. 2a, b). Serum β-d-glucan levels were within the normal range until POD 6, but started to increase on POD 12 (Fig. 1). Bronchoalveolar lavage revealed microscopic filamentous fungus on POD 13. Micafungin was administered, followed by voriconazole (assuming aspergilloma); subsequent chest computed tomography (CT) showed right middle lung lobe infiltration. Surgery was planned since the lesion continued to grow (Fig. 2b).

Preoperative examination revealed decreased ejection fraction (40%) on echocardiography and ST elevation on electrocardiography. There was no dominant stenosis on cardiac arteriography on POD 19.

Right lung segmentectomy was performed on POD 20 (Fig. 2c, d). *C. bertholletiae* was identified by polymerase chain reaction (PCR) from operative specimens cultured using Sabouraud dextrose agar culture medium (BD, Fukushima, Japan) (Fig. 2e–g). β-d-glucan level elevated five times the upper limit of the normal range (60.93 pg/mL), and amphotericin B (AmB) was started at 5 mg/kg IV on POD 22 (Fig. 1).

**Fig. 3:**
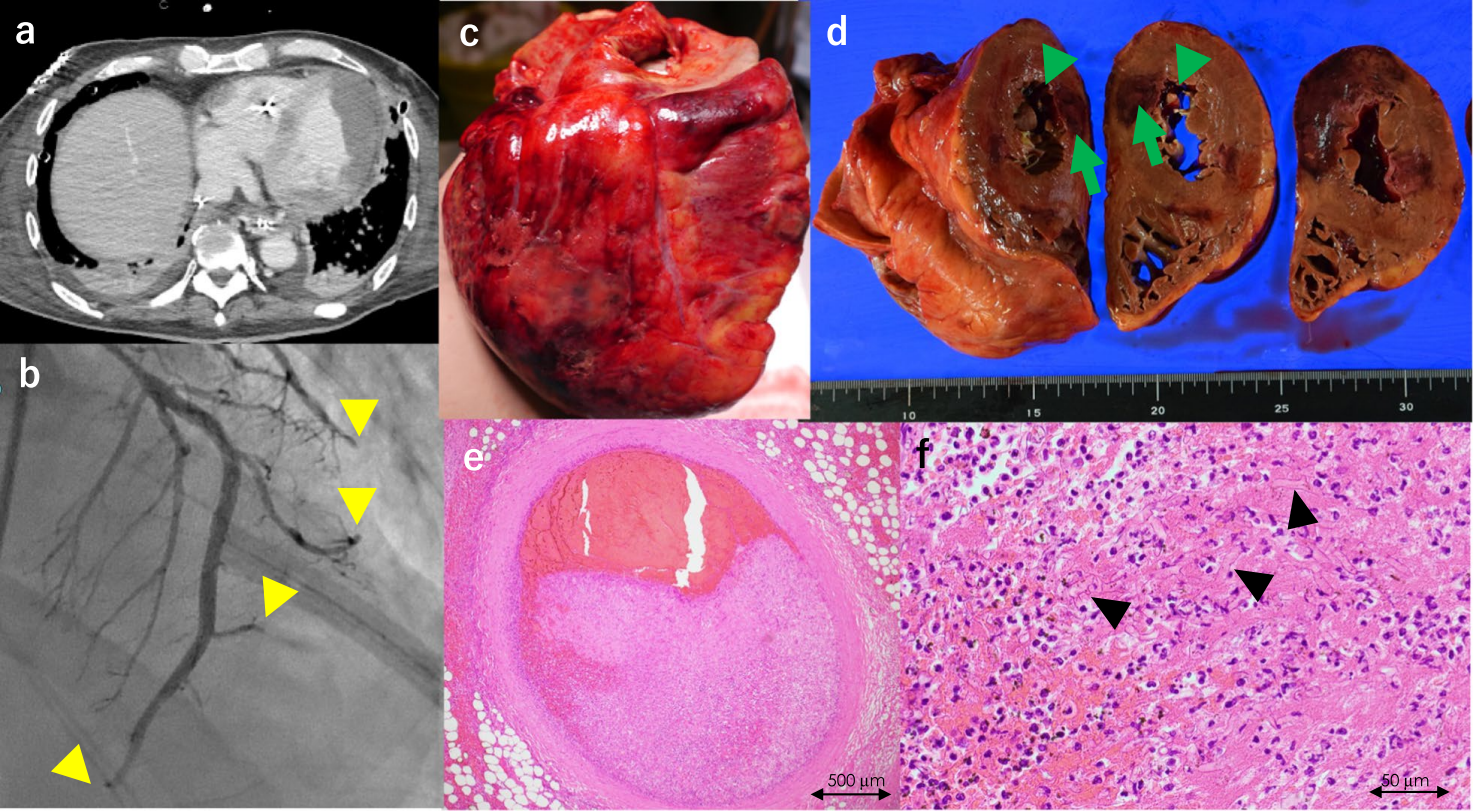
Heart lesions. **a** Enhanced CT reveals an unenhanced area in the heart wall, suggesting multiple myocardial infarctions on POD 27. **b** Percutaneous cardiogram showing non-stenotic coronary arteries with multiple peripheral disruptions (yellow arrowheads). **c**–**f** Autopsy; multiple myocardial infarctions are penetrating the whole wall of the heart (green arrow). Vegetation is formed along with the chordae tendineae in the left ventricle (**d**, green arrowheads). Coronary arteries are patent but filled with fungal thrombosis on the peripheral side (**e**). Fibrin-containing fungal hyphae (arrowheads) cover the endothelium in all chambers, indicating fungal endocarditis (**f**). *CT* computed tomography, *POD* postoperative day

**Fig. 4:**
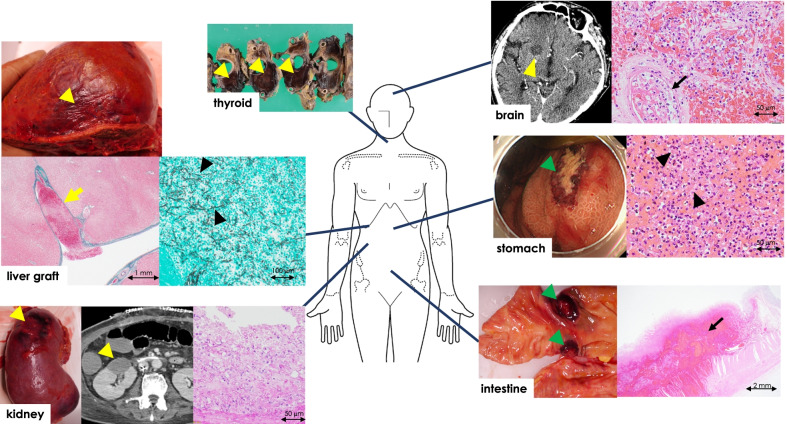
Autopsy findings. The brain, thyroid, liver graft, and kidney have infarctions (yellow arrowheads). A thrombus filled with fungal hyphae stained by Grocott is detected in a branch of the portal vein in the liver graft (yellow arrow). The stomach and intestine have multiple ulcers (green arrows), pathologically represented as bleeding necroses. Many fungal hyphae (arrowheads) are seen in atrial thrombosis (arrows) and tissues of the brain, kidney, stomach, and intestine

On POD 23, the patient’s circulatory dynamics collapsed, and he developed bigeminy and atrial-ventricular block, thus requiring a temporary pacemaker. Enhanced CT scans revealed multiple myocardial (Fig. 3a), cerebral (Fig. 4), and renal (Fig. 4) infarctions on POD 27. Cardiac arteriography revealed multiple peripheral occlusions (Fig. 3d). The patient’s cardiac arterial occlusion did not resolve despite percutaneous coronary intervention. An intra-aortic balloon was inserted for circulatory support; however, the recipient died of cardiac failure on POD 28.

Hypothrombocytopenia was present since the pre-transplant period, which worsened after the LDLT. The patient’s bone marrow had reduced megakaryocytes and a pseudo-Pelger–Huët anomaly on POD 16. The recipient was diagnosed posthumously with myelodysplastic syndrome due to hereditary abnormalities.

Autopsy revealed multiple microscopic bleeding infarctions in the brain (Fig. 4), thyroid (Fig. 4), heart (Fig. 3), lung (Fig. 2), liver (Fig. 4), gastrointestinal tract (Fig. 4), and kidneys (Fig. 4). The cardiac arteries were patent but filled with fungal thrombi on the peripheral side. A fibrin-containing fungal mass covered the endothelium in all chambers, suggesting fungal endocarditis (Fig. 2d–f). Other organs also had multiple bleeding infarctions due to arterial septic emboli caused by fungi.

## Discussion

Mucormycosis is caused by various genera of the order *Mucorales* of the class *Zygomycetes*, and the terms mucormycosis and zygomycosis are often used interchangeably [7]. *Cunninghamella* can also cause infection in humans, which affect multiple organ systems, including the rhinocerebral, pulmonary, cutaneous, and gastrointestinal systems, and cause severe infections [8]. In this case of mucormycosis after LDLT, the brain, lung, heart, gastrointestinal tract, liver, and kidneys were involved, and progression was rapid.

Mucormycosis is uncommon in developed countries [9]. Diabetes mellitus is the most common risk factor, followed by hematologic malignancies and solid organ or hematopoietic cell transplantation [9]. A review of mucormycosis in 116 organ transplant patients found that mucormycosis was caused by *C. bertholletiae* only in 6% of the cases [10]. To the best of our knowledge, only one case of *C. bertholletiae* invading multiple organs after liver transplantation has been reported since 1988 [11], wherein the diagnosis of *C. bertholletiae* infection was obtained only by a microscopic finding in autopsy; however, there were no representative images of multiple organ dissemination. Our study is the first to report detailed information, including several images on mucormycosis with multiple organ dissemination caused by *C. bertholletiae* after liver transplantation.

The initial right lung lesion was resected and used to diagnose mucormycosis. However, by that time, other symptoms such as altered consciousness and decreased ejection fraction rate (probably associated with the cerebral and cardiac invasions) had appeared. Pulmonary lesions are common in immunocompromised patients, including solid organ transplant recipients with mucormycosis [4, 10, 12]. *Mucorales* are ubiquitous in nature, and infection usually begins in a nasal turbinate or alveolus in susceptible individuals [9]. Pulmonary and rhino-orbital-cerebral mucormycosis are acquired by spore inhalation. Mucormycosis develops after a median of 5 months following solid organ transplantation, with a significantly earlier occurrence in liver transplant recipients [13]. Mucormycosis agents such as *C. bertholletiae* penetrate vasculature, resulting in mycotic thrombi, which can cause infarction with hemorrhagic necrosis [9]. Our case confirmed that the pulmonary lesion pathologically contained hemorrhagic necrosis caused by fungal emboli.

Cardiac mucormycosis caused Takotsubo myocarditis and an ultimately fatal cardiac infarction. Multiple myocardial infarctions due to fungal emboli were pathologically proven in autopsy specimens. The myocardia were most commonly affected in patients with disseminated pulmonary mucormycosis following solid organ transplantation [10].

Autopsy also revealed fibrin-containing hyphae covering the endothelium in all heart chambers, suggesting fungal endocarditis. Multiple organ infarctions occurred in quick succession; dissemination to multiple organs may have occurred originating from fungal endocarditis. Zhang et al. reported a case of endocarditis caused by *C. bertholletiae* infection after kidney transplantation [14].

Previous solid organ transplantation was associated with an increased risk of pulmonary, gastrointestinal, or disseminated infection in patients with mucormycosis [15]. An underlying hematological malignancy increases the risk of organ dissemination [3, 15], which occurs more frequently in the distant organs of immunosuppressed patients [10]. Our patient had infection-promoting factors, such as organ transplantation, bone marrow dysfunction, and immunosuppression. Prophylaxis might be needed against fungal infection, especially in the case of induced immunosuppression before transplantation, although the selection of anti-fungal reagents is complicated.

Previous use of voriconazole is a significant risk factor for mucormycosis development [16, 17]. We transitioned the patient to voriconazole when an *Aspergillus* infection was suspected before diagnosing mucormycosis. Jeong et al. reported that prior antifungal prophylaxis, including voriconazole and fluconazole, was administered to 11% of patients with mucormycosis [3]. Dissemination might have expanded to include multiple organs during the administration of voriconazole.

The risk of mortality is approximately 11-fold higher in patients with disseminated mucormycosis than in those with localized disease [4]. *Mucorales* were grown on culture, and histopathological features contributed to a definitive diagnosis of mucormycosis [3]. The presence of broad, aseptate, or pauci-septate hyphae with wide-angle branching with tissue invasion provides evidence of mucormycosis [18]. However, early diagnosis of *C. bertholletiae* infection is difficult and may only be confirmed on autopsy [14]. Modern molecular-based methods are required for species identification and diagnosis because serologic tests are not available yet [19]. We suspected mucormycosis after culturing the operative specimen; *C. bertholletiae* was ultimately identified by PCR, detecting specific fungal DNA sequences.

Patient mortality increases if AmB-based therapy is delayed [20]. High-dose conventional AmB at a dose of 10 mg/kg/day for 6 months was reported to contribute to the successful treatment of disseminated mucormycosis after stem cell transplantation [21]. Surgical intervention with debridement of necrotic tissue and debulking improves survival in patients with pulmonary infections [12]. Surgery plus AmB produces superior results than those of AmB administration alone [5]. Discontinuation or reduction in immunosuppression was associated with a better survival rate in patients after solid organ transplantation [10]. AmB was administered after removing the pulmonary lesion; however, the treatment was not completed in our patient because of brain and heart involvement. AmB should be administered as quickly as possible to LDLT recipients with suspected fungal infection.

## Conclusions

Mucormycosis caused by *C. bertholletiae* after liver transplantation is a rare but fatal complication; the subsequent organ invasion can be fast and aggressive. Early diagnosis and appropriate treatment using antifungal agents and surgery are necessary to prevent dissemination to multiple organs.

## Data Availability

All data generated or analyzed during this study are included in this published article.

## Abbreviations

AmB: Amphotericin B
CT: Computed tomography
HCC: Hepatocellular carcinoma
LDLT: Living donor liver transplantation
MMF: Mycophenolate mofetil
PCR: Polymerase chain reaction
POD: Postoperative day
